# Addressing men's health policy concerns in Australia: what can be done?

**DOI:** 10.1186/1743-8462-4-20

**Published:** 2007-10-10

**Authors:** James A Smith

**Affiliations:** 1 Department of Paramedic & Social Health Sciences, Flinders University, Adelaide, South Australia, Australia; 2 Discipline of Public Health, University of Adelaide, Adelaide, South Australia Australia

## Abstract

There is a lack of consensus about what *men's health *constitutes in Australia. The absence of a widely accepted definition has been problematic for establishing state and national men's health policies. I consider that one impediment to the implementation of state and federal men's health policies has been a lack of willingness to approach men's health from a broad public health perspective. In particular, scant attention has been paid to exploring lay perspectives of how men define and understand health, and in turn, how these relate to significant policy problems such as men's health service use. I conclude by suggesting that a focus on men's lay perspectives of their health emerging from the United Kingdom and the Republic of Ireland provides a useful framework to guide men's health policy discussion in Australia.

## Background

### Defining men's health as a policy problem

In Australia professional interest in men's health has grown markedly over the past decade [1]. The last two years alone has seen both the Australian Medical Association and the Royal Australian College of General Practice release position statements relating to men's health [2,3]. The *Medical Journal of Australia *even dedicated a special edition to men's health in October 2006. While there are inconsistent ways of defining men's health, a common concern raised in almost all recent scholarship relating to men's health in Australia is the lack of commitment to developing and implementing men's health policies at state and federal levels [1-6]. This concern is best summarised by Greg Malcher the National Convenor of GPs4Men who claims:

*Australia still has no national men's health policy, despite the existence of a women's health policy since 1989. It would be naïve to suggest that simply developing a policy would be sufficient to deal with all the challenges of men's health – policy without adequately funded programs = "piffle". Yet, for those of us involved in men's health, there remains an overwhelming desire to see a formal acknowledgement by the federal government (whether a policy, position statement or other document) of the broad and unique issues of men's health, and a preparedness to fund a national program to address these issues*. [6]

Currently, New South Wales is the only state in Australia to have succeeded in producing a men's health policy document that has been endorsed by a state Health Minister [5,7]. It is worth noting, however, that this document – *Moving Forward in Men's Health *– was never explicitly labelled as a policy. This is a clear indication of the reluctance to use the word policy in the context of men's health. So why is there a reticence to implement state and national men's health policies in Australia and what can be done to rectify this significant public health concern?

Firstly, debates relating to men's health policy development in Australia are not new. Indeed, there has been ongoing policy discussion at state and federal levels for quite some time [8-10]. A review of relevant literature reveals that many draft men's health policy documents have been developed during the past two decades, but that there have been major impediments to their formal endorsement and subsequent implementation [10]. There are four broad issues relating to the preclusion of men's health policy from state and federal agendas in Australia. These relate to medical dominance, the lack of a men's social movement, the Australian political and policy climate, and aspects of Australian men's culture [10]. Other commentators have argued that the lack of a well articulated theoretical orientation to direct men's health policy development has been problematic [11]. Divergent, narrow or inadequate definitions of men's health have also hindered policy responses [5,10]. It is this latter concern that I discuss in this paper.

## Discussion

### Defining men's health: A policy problem

'Men's health' is a term frequently used by the media, academics, health practitioners and the general public. However, there are subtle differences between how men's health can, or should, be defined [5,10,12,13]. While there is a wide recognition that men's health extends beyond male-specific conditions of the reproductive organs, such as prostate problems, testicular concerns and erectile dysfunction, these concerns have remained a prominent feature of the international discourse relating to men's health [5]. Epidemiological data has also been a central feature of men's health commentary, with comparisons between the status of men's and women's health predominating this discussion [10]. These definitions have contributed to a broader conceptualisation which suggests that men's health is perceived as being akin to a disease or condition unique to men, more prevalent in men, more serious among men, for which risk factors are different for men or for which different interventions are required for men [14]. Yet, even this definition has its limitations.

More recent commentary has drawn attention to the usefulness of understanding men's health in relation to social and economic determinants of health [1,5]. As such, health equity has become a central focus of this contemporary men's health discourse, where an emphasis has been placed on shifting resources towards the most vulnerable and disadvantaged groups of men [1,5]. Other considerations which complicate efforts to define men's health have also emerged, such as the burgeoning body of research relating to hegemonic masculinity and multiple masculinities [1]. At this juncture it is worth considering what this definitional dilemma means for men's health policy development in Australia. Collectively these understandings of men's health raise concern over whether men's health policy discussion should be focused on mainstream men's health issues – where all men are perceived to be the same, vulnerable groups of men – where variation between men is acknowledged, or perhaps both?

Irrespective of the policy approach advocated, the above evidence clearly demonstrates that a broader, more appropriate view of men's health is needed to develop a comprehensive national policy [4,5,15]. One way of embedding a broader conceptualisation of men's health into the current policy discussion, and one which has remained almost entirely absent from this discussion to date, is the inclusion of lay perspectives of health. There is no academic scholarship originating in Australia, of which the author is aware, that specifically links men's lay perspectives of their health with key policy concerns relating to men's help seeking practices, health service use and the way in which men navigate the current health system

### Closing the gap: Lay knowledge in men's health policy discussion

Public health commentators have argued that there is a need to move beyond traditional forms of scientific knowledge to guide development of both healthy public policy and local public health programs [16-18]. In particular, previous commentary has shown that lay perspectives are particularly useful in understanding and addressing significant public health concerns [17,19-22]. Moreover, qualitative studies exploring lay knowledge are considered to be more persuasive in influencing policy makers than expert knowledge [18]. When there are differences in perspective among stakeholders in how to address particular health issues, as is the case in men's health, there is a need to explore the interface between professional and community understandings to maximise potential health gains [23,24]. A criticism of the discourse employed by health professionals about men's health – particularly that associated with hegemonic masculinity – has been the perpetuation of a 'men behaving badly' stance [5,25]. The inclusion of lay perspectives of men's health increases the capacity to move beyond this male-deficit model by providing an opportunity to understand men as real people, who live, work and play within multiple communities [1,5,25-28].

To provide a more persuasive men's health policy argument in Australia, and to facilitate a broader conceptualisation of what men's health constitutes, male consumer viewpoints ought to be considered when describing men's health. Yet, specific empirical data on male lay perspectives of health and well-being have largely remained absent in research on men's health [22], and this has been a contributing factor that has stalled the development and implementation of men's health policy in Australia [10]. Indeed, successes in women's health policy development in Australia have arisen out of a political discourse that has paid particular attention to women's lived experiences [10]. While it would seem sensible to conduct such research with men, there has been limited stimulus to determine men's understandings of health and well-being in Australia [10]. Yet, this has not been the case in other parts of the world.

There is a growing body of public health research emerging from the Republic of Ireland, Scotland and England which has shown an appreciation of lay perspectives of men's health [22,26,29]. This has assisted in understanding how 'health' is conceptualised differently between marginalised groups of men, such as gay men and disabled men. More importantly this has been used to describe their differential use of health services [22,27]. For example, the way in which men interpret, and respond to their chest pain [26], or the way in which men conceptualise their health, particularly in relation to risk [22,29]. While there is little evidence of the effective translation of this research into policy discussion, potential exists to do so. Interestingly, gender sensitive care in Britain has been supported by a policy emphasis on the importance of eliminating inequalities in the provision of health care, which intersects with this type of exploratory research [27,30]. Likewise, men's health discussion papers considered to be precursors to the development of a men's health policy in the Republic of Ireland have also paid attention to the ways men define certain aspects of their health [29].

## Conclusion

The aim of improving the health status of men should, undoubtedly, be focused on developing valid and reliable data on men's perceptions of their health, their health practices and their health needs [31]. More importantly this data must be used to advocate for, and frame, emerging men's health policy responses in Australia. Of course, there are other considerations such as financial constraints and shifting timescales that influence this approach [32]. However, it is time for Australian men's health researchers, practitioners and policy makers to consider the achievements of their colleagues in the UK and the Republic of Ireland to adopt a consumer-focused public health response to develop and implement a national men's health policy here in Australia. Political will is required to make this happen.
